# Blasting Into Acute Coronary Syndrome

**DOI:** 10.7759/cureus.19589

**Published:** 2021-11-15

**Authors:** Swetha R Nuthulaganti, Fadi Kandah, Marcos E Valentin, Maria Gutierrez

**Affiliations:** 1 Internal Medicine, University of Florida College of Medicine, Jacksonville, USA

**Keywords:** non-st-elevation myocardial infarction, clinical case report, left heart catheterization, nstemi, acute coronary syndrome, blast crisis, chonic myelogenous leukemia

## Abstract

Acute coronary syndrome (ACS) is a condition that develops from reduced blood flow and oxygen delivery through the coronary arteries which leads to cardiac ischemia. In the case presented here, the patient’s ACS was precipitated by his underlying condition of chronic myelogenous leukemia (CML). Several complications can arise in patients with CML, one of them being blast crisis. Blast crisis is defined by 20% or greater blasts in the peripheral blood, or extramedullary proliferation of blasts.^ ^There is a known phenomenon of blood hyperviscosity that can develop in such patients which can lead to complications of stroke-like symptoms, congestive heart failure, and acute respiratory failure. In such cases, leukostasis rarely leads to myocardial ischemia. We present a challenging case of a patient with an acute coronary syndrome (ACS) precipitated by a blast crisis. This case highlights a potentially life-threatening cardiac complication of CML in patients with coronary artery disease and aimed to provide an optimal treatment strategy to improve outcomes.

## Introduction

When patients initially present with chest pain, a diligent workup is required to rule out cardiac chest pain. As with any acute coronary syndrome (ACS) case, the typical workup includes obtaining an EKG and troponins. In cases of ST-elevation myocardial infarction, the ischemia is caused by an acute occlusion in the coronary vessel resulting in transmural ischemia, myocardial injury, or necrosis [1]. In cases where the patient presents with ST-elevation myocardial infarction (STEMI), the patient is immediately taken to the cath lab for revascularization and percutaneous intervention. However, a non-ST-elevation myocardial infarction (MI) is often more complicated with diagnosis and treatment. A non-ST-elevation myocardial infarction (NSTEMI) occurs when there is partial or intermittent occlusion of the coronary vessels causing subendocardial ischemia. In order to properly treat patients with NSTEMI, a distinction between NSTEMI and type II MI must be made. In patients with type II MI, troponins are elevated secondary to physiological causes that are not cardiac in origin. Type II myocardial infarction does not involve an unstable plaque but rather affects areas of chronic stenosis under physiologic stress [2]. Patients who present with NSTEMI often have recurrent events and worse outcomes. In cases where ACS is complicated by chronic myelogenous leukemia (CML) and other hematologic disorders, the prognosis is often worse.

This study was previously presented as a poster in the Florida Chapter American College of Physicians (ACP) Spring 2021 Virtual Poster Competition on March-April 2021. (https://www.acponline.org/system/files/documents/about_acp/chapters/fl/2021_florida_chapter_abstract_instructions.pdf)

## Case presentation

A 72-year-old male with a history of coronary artery disease (last left heart catheterization completed in Puerto Rico several years ago reportedly revealing obstructive coronary artery disease {CAD}), chronic myelogenous leukemia (diagnosed in 2020), and hypertension (controlled on isosorbide mononitrate 30 mg, metoprolol succinate 25 mg) presented with complaints of shortness of breath and typical chest pain. The patient described the chest pain as nonpositional, nonreproducible, nonradiating chest tightness that waxes and wanes. He stated that his symptoms are worsened with exertion and relieved with rest. He denied experiencing symptoms like this in the past and reports being compliant with his prior medication. On presentation, the patient was noted to have tachycardia with a heart rate of 102 beats per minute, diaphoretic, and was hypoxic (SpO2 of 89%) requiring 2 L of nasal cannula.

**Figure 1:**
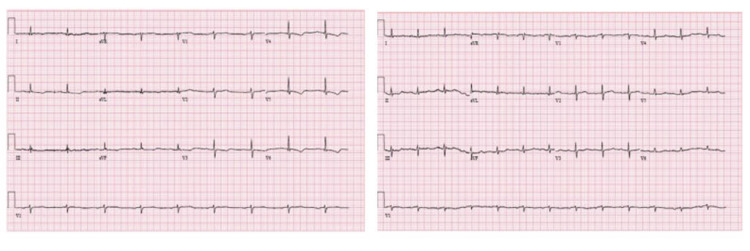
EKG at presentation with T wave inversions in leads V3-V6 (left) and EKG after treatment of hyperleukocytosis with resolution of T wave inversions (right)

Electrocardiogram (EKG) was significant for sinus tachycardia and T wave inversion in the lateral leads (V3-V6) which were new from the patient’s prior EKG. Labs were significant for an elevated troponin, initial troponin T of 1.32 ng/mL that up-trended to 1.56 ng/mL, creatine kinase myocardial band (CKMB) was noted to be 15.5 ng/mL and up-trended to 43.5 ng/mL within the next hour. Due to concerns for ACS, the patient was started on a heparin drip for non-ST-elevation myocardial infarction (NSTEMI) (Figure 1).

Complete blood count (CBC) showed a significant elevation of white blood counts (WBC) at 106,000/uL, with predominantly neutrophils upon differentiation. The remainder of the CBC was significant for hemoglobin of 12.0 g/dL, hematocrit of 40.0%, MCV of 109.0 fL, and RDW of 14%. The patient's chest x-ray was significant for diffuse bilateral interstitial changes with faint hazy ground-glass opacity in the left mid to lower lung. The respiratory viral panel was significant for adenovirus, remainder of infectious workup was negative. The patient's echocardiogram was notable for an ejection fraction of 45-50%, with anteroseptal akinesis, inferoseptal hypokinesis, apical akinesis, and elevated left ventricular filling pressures. The right ventricle and right atrium were noted to be mildly enlarged.

Emergent plasmapheresis was considered due to concerns for blast crisis as the precipitant for the patient’s acute coronary syndrome. However, due to the patient’s pre-existing coronary artery disease, it was determined to defer this therapy and opt for a more invasive approach to prevent continued ischemia and myocardial death. The patient was treated with intravenous fluids and administered hydroxyurea to reduce leukostasis and proceed safely with coronary angiography.

**Figure 2:**
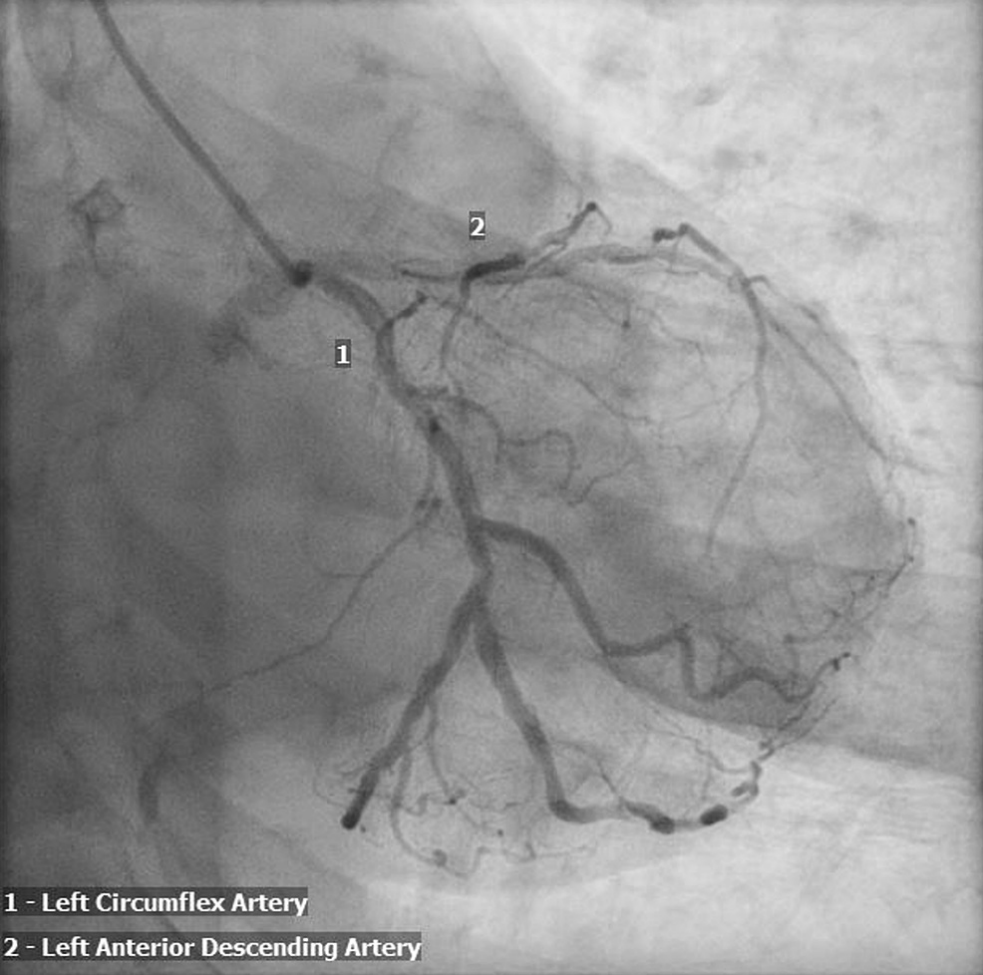
Left heart catheterization demonstrating moderate nonobstructive coronary artery disease mainly in the left anterior descending coronary artery

**Figure 3:**
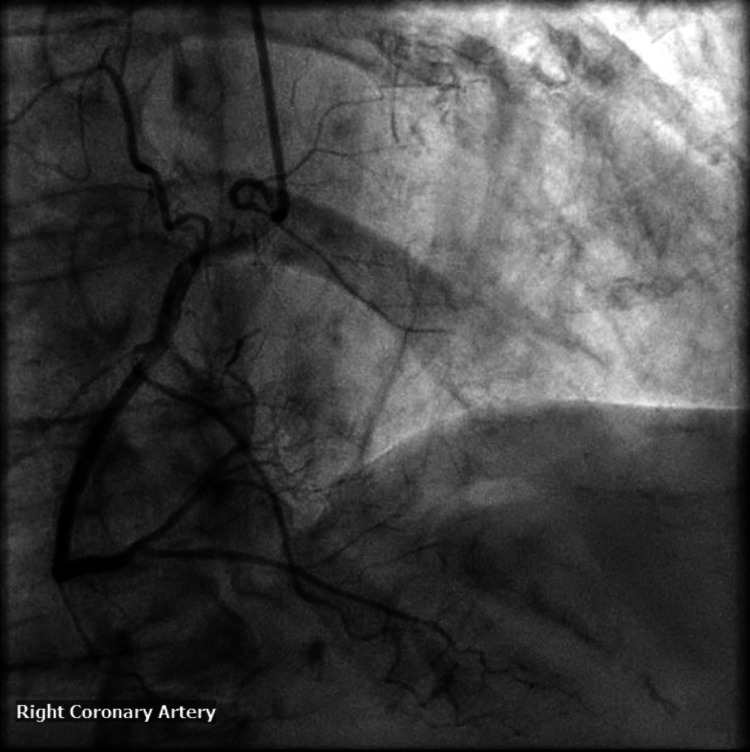
Left heart catheterization of the right coronary artery demonstrating nonangiographically significant coronary artery disease

Coronary angiography revealed nonobstructive left anterior descending artery disease, no record of the patient's previous left heart catheterization to compare (Figures 2, 3). There were no obvious culprit lesions to undergo percutaneous intervention, and the patient was managed medically with aspirin 81 mg, clopidogrel 75 mg, and rosuvastatin 20 mg.

**Figure 4:**
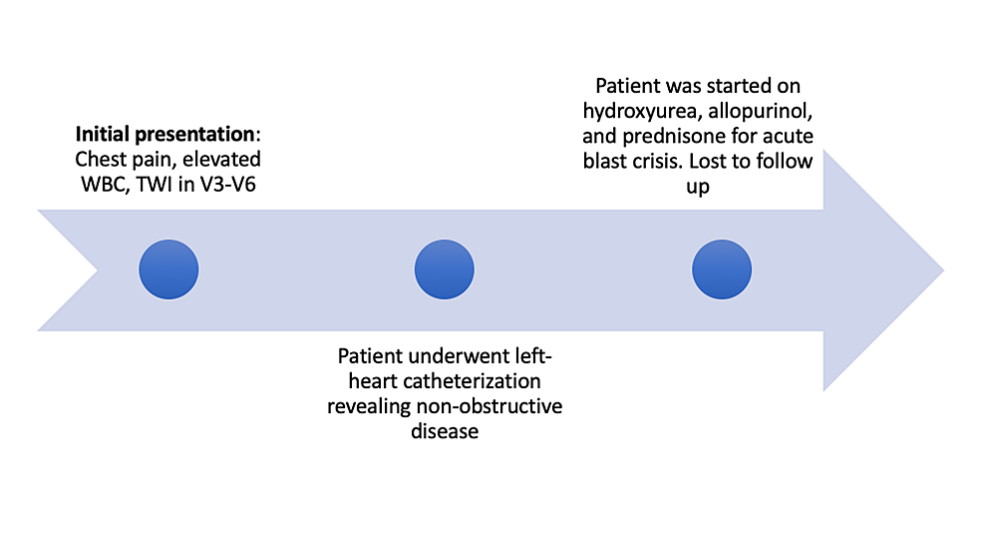
Timeline of events

The patient's white blood count decreased to 79,000/uL with intravenous fluids and prednisone. The patient had no further episodes of chest pain and repeated EKGs after correction of leukocytosis showed a resolution of the previous T wave inversions. The patient was discharged in stable condition after completing treatment with hydroxyurea, allopurinol, and a course of prednisone for his acute blast crisis. The patient was recommended to follow up with cardiology, however, the patient was lost to follow up. Below is a timeline summarizing the events of the patient's clinical care (Figure 4).

## Discussion

Hyperleukocytosis, defined as a white blood cell (WBC) count of greater than 100,000/uL, increases the propensity of vascular occlusions by intensifying the viscosity of blood. This phenomenon is of particular significance in patients with chronic myelogenous leukemia (CML) where there is progression from a chronic, indolent stage of disease to an accelerated stage of proliferation, followed by a terminal stage known as blast crises [3]. Blast crisis is recognized as a rapid expansion and differentiation of myeloid or lymphoid cells. The primary insult involves the translocation of BCR-ABL protein ultimately causing an increase in tyrosine kinase activity [4]. The prognosis of patients with blast crisis has been shown to be directly related to the number of blast cells. Patients with greater than 30% blasts have been shown to have increased morbidity and mortality.

Patients who develop blast crisis are at risk of life-threatening bleeding or ischemia. The brain and lungs are primarily affected but cardiac complications are also of concern. Patients may develop acute coronary syndrome and progressive myocardial ischemia adding to the complexity of care. When blast crisis is identified, patients should undergo emergent medical therapy to control the increase in blast cells [5]. Pharmacologic therapy for CML typically involves the use of hydroxyurea, busulfan, or interferon-alpha [4].

The concurrent presentation of blast crisis with an ACS clouds the picture of which to primarily treat. As seen in the case presented above, patients with co-morbidities of coronary artery disease with CML have a predisposition for worsening cardiac ischemia in areas of prior stenosis. Clinicians should have a high suspicion of ACS in these patients who develop typical chest pain symptoms in the setting of known coronary artery disease. Percutaneous coronary intervention (PCI) can be used to treat stenotic vessels and reduce further ischemic damage to the heart. It is a potentially life-saving intervention and should not be delayed in high-risk patients [6]. Dual antiplatelet therapy (DAPT) and anticoagulation are also typically utilized under the premise of reducing thrombus formation by inhibiting both platelet aggregation and fibrin deposition [6]. Caution should be exercised however when using DAPT, given the conundrum of hypercoagulability and platelet dysfunction seen in patients with blast crises.

Neutrophils have been found to play a role in unstable atherosclerotic plaques suggesting a relationship between diseases causing systemic inflammation and ACS. The pathophysiology may be related to the neutrophilic production of enzymes such as elastase and myeloperoxidase [7]. One particular study assessed 573 people who underwent coronary angiography and determined that there is a correlation between the number of WBCs and the severity of CAD with an r-value of 0.16 and p-value of 0.0001 [8]. Those with elevated white cell counts have also been shown to have an increased risk of re-infarction [6].

In our case, the patient had a known history of coronary artery disease leading to an increased risk of vascular occlusion in the setting of hyperviscosity from his CML. In addition to causing hyperviscosity, blast cells compete aggressively with normal organ tissue for oxygen consumption. Further microvascular occlusion may be propagated by white cell plug formation [9]. In particular, patients with nonobstructive coronary artery disease are at increased risk for ischemia in the setting of blast crisis. This case demonstrates the rare findings of an NSTEMI in a patient with underlying non-obstructive CAD, propagated by the hyperviscosity of his blast crisis. While most leukemic patients with blast crisis benefit primarily from reducing the hyperviscosity associated with extreme leukocytosis, this case demonstrates the importance of considering early PCI and initiation of antiplatelet therapy in patients with predisposing factors for myocardial injury who present with ACS.

## Conclusions

This patient had good outcomes with a more cardiac-focused invasive approach to management. Those with known coronary artery disease may have better results with coronary angiography prior to initiation of treatment for blast crisis. Further research is warranted to provide definitive guidelines on the management of those with these overlapping syndromes.

Areas of coronary obstruction can potentially propagate myocardial ischemia for patients in blast crisis. Those with known CAD may have better results following coronary angiography to unveil underlying coronary anatomy prior to initiation of treatment for blast crisis. Early Intervention in these areas of obstruction in patients with CML may improve outcomes if done prior to the development of blast crisis. This patient had good outcomes with a more cardiac-focused invasive approach to management. Further research is warranted to provide definitive guidelines on the management of those with these overlapping syndromes.

## References

[1] Akbar H, Foth C, Kahloon RA, Mountfort S (2021). Acute ST elevation myocardial infarction. StatPearls [Internet].

[2] Collinson P, Lindahl B (2016). Diagnosing type 2 myocardial infarction. Am Coll Cardiol.

[3] Calabretta B, Perrotti D (2004). The biology of CML blast crisis. Blood.

[4] Enright H, McGlave PB (1996). Chronic myelogenous leukemia. Curr Opin Hematol.

[5] Lauseker M, Bachl K, Turkina A (2019). Prognosis of patients with chronic myeloid leukemia presenting in advanced phase is defined mainly by blast count, but also by age, chromosomal aberrations and hemoglobin. Am J Hematol.

[6] Jao GT, Knovich MA, Savage RW, Sane DC (2014). ST-elevation myocardial infarction and myelodysplastic syndrome with acute myeloid leukemia transformation. Tex Heart Inst J.

[7] Naruko T, Ueda M, Haze K (2002). Neutrophil infiltration of culprit lesions in acute coronary syndromes. Circulation.

[8] Kostis JB, Turkevich D, Sharp J (1984). Association between leukocyte count and the presence and extent of coronary atherosclerosis as determined by coronary arteriography. Am J Cardiol.

[9] Katogiannis K, Ikonomidis I, Panou F (2018). Leukostasis-related fatal cardiopulmonary arrest as initial chronic myeloid leukemia presentation. J. Med. Cases.

